# The size diversity of the Pteridaceae family chloroplast genome is caused by overlong intergenic spacers

**DOI:** 10.1186/s12864-024-10296-0

**Published:** 2024-04-22

**Authors:** Xiaolin Gu, Lingling Li, Xiaona Zhong, Yingjuan Su, Ting Wang

**Affiliations:** 1 College of Life Sciences, South China Agricultural University, 510642 Guangzhou, China; 2 School of Life Sciences, Sun Yat-sen University, 510275 Guangzhou, China; 3 Research Institute of Sun Yat-sen University in Shenzhen, 518057 Shenzhen, China

**Keywords:** Pteridaceae, Chloroplast, Evolutionary genomics, Structural comparison, Divergence time

## Abstract

**Background:**

While the size of chloroplast genomes (cpDNAs) is often influenced by the expansion and contraction of inverted repeat regions and the enrichment of repeats, it is the intergenic spacers (IGSs) that appear to play a pivotal role in determining the size of Pteridaceae cpDNAs. This provides an opportunity to delve into the evolution of chloroplast genomic structures of the Pteridaceae family. This study added five Pteridaceae species, comparing them with 36 published counterparts.

**Results:**

Poor alignment in the non-coding regions of the Pteridaceae family was observed, and this was attributed to the widespread presence of overlong IGSs in Pteridaceae cpDNAs. These overlong IGSs were identified as a major factor influencing variations in cpDNA size. In comparison to non-expanded IGSs, overlong IGSs exhibited significantly higher GC content and were rich in repetitive sequences. Species divergence time estimations suggest that these overlong IGSs may have already existed during the early radiation of the Pteridaceae family.

**Conclusions:**

This study reveals new insights into the genetic variation, evolutionary history, and dynamic changes in the cpDNA structure of the Pteridaceae family, providing a fundamental resource for further exploring its evolutionary research.

**Supplementary Information:**

The online version contains supplementary material available at 10.1186/s12864-024-10296-0.

## Introduction

Ferns are one of the oldest and most primitive vascular plant groups on Earth [1]. They are a group of vascular plants with independent gametophyte and sporophyte generations, mainly undergoing sexual reproduction through spores. Pteridaceae is the second most genera-rich fern family. According to the Pteridophyte Phylogeny Group I (PPG I) classification, Pteridaceae contain five subfamilies, 53 genera, with an estimated 1,211 species contributing to about 10% of extant leptosporangiate fern diversity [2, 3]. These species have multiple values. For example, the *Pteris* species can accumulate arsenic, which is of great significance for the remediation of heavy metals in soil [4]. Plenty of the *Adiantum* species can be used in medicine and are used in different parts of the world [5–8]. Pteridaceae species have a cosmopolitan distribution concentrated in wet tropical and arid regions, occupying various ecosystems such as terrestrial, epiphytic, rupestral, and even aquatic [9].

In plants, chloroplasts are the site of photosynthesis and play an important role in the synthesis of defense-related hormones, which sustain life on Earth [10, 11]. Chloroplasts also participate in some metabolic processes [11] and play important roles in plant adaptation to environmental stress [12–14]. Chloroplasts typically possess independent genes and mechanisms for gene expression [15]. The chloroplast genomes (cpDNAs) of land plants are typically 110–160 kb in size [16], usually divided into a large single-copy (LSC) region and a small single-copy (SSC) region by a pair of inverted repeats (IRa and IRb), forming a typical quadripartite structure [17]. The cpDNA is mostly inherited from one parent, its structure is conservative, recombination is less, and the substitution rate is much lower than that of the nuclear genome [18–20]. With the advancement of sequencing technology, cpDNA has become more accessible, therefore, it has become increasingly common to use chloroplasts to explore plant evolutionary events [21, 22].

Comparing complete cpDNAs contributes to the study of mechanisms underlying genome evolution, revealing evolutionary relationships and phylogenies among species. For instance, lycophytes share a similar chloroplast gene order with mosses, while displaying an inverted gene order compared to all other vascular plants, providing evidence for the ancient evolution of early vascular land plants [23]. Similarly, the expansion and contraction of the IR region often serve as evidence for interspecies phylogenetic relationships in chloroplast genome studies [24, 25]. In the context of evolution, the substantial loss of genes was initially linked to endosymbiotic events, but subsequent research indicates that gene loss independently occurred in different lineages [26]. This suggests that a series of complex evolutionary constraints, selection, and convergence led to the conservation of chloroplast genome structure and content. For instance, the evolution of parasitic angiosperms has resulted in the relaxation of evolutionary constraints associated with the maintenance of photosynthetic functionality. Therefore, in the early stages of parasitic evolution, some photosynthetic genes (such as *ndh*-) were lost, leading to significant changes in chloroplast genome content [27].

Research has shown that the size of cpDNA is often influenced by changes in the IR boundaries [28]. Furthermore, a high number of repetitive sequences in cpDNA has been recognized as a contributing factor to variations in genome size [29–31]. In this study, the variations in sizes of Pteridaceae cpDNAs were ascribed to alterations in the length of overlong intergenic spacers (IGSs), with these IGSs exhibiting species-specific differences. Through sequencing the cpDNAs of five Pteridaceae species and comparing their structures with 36 other reported cpDNAs in the Pteridaceae family, this study aimed to uncover the evolutionary dynamics, genetic variations, and evolutionary relationships of cpDNAs among different species within the Pteridaceae family.

## Results

### Basic characteristics of Pteridaceae

The sizes of the Pteridaceae cpDNAs in this study ranged from 145,327 bp to 165,631 bp, with a GC content varied from 36.7% to 45.3%. They all possessed typical quadripartite structures, of which the LSC region was 80,810 − 89,030 bp, the SSC region was 19,930 − 27,974 bp, and the IR regions were 42,054 − 61,842 bp (Table 1). The accuracy of gene annotations for these 41 Pteridaceae species was rechecked, using the reference sequences of *Adiantum capillus-veneris* and *Adiantum shastense*, and missing annotations were supplemented by conducting local BLAST to retrieve homologous sequences (Figure S1). The statistics of lost chloroplast genes showed that *Paragymnopteris bipinnata* var. *bipinnata* and *Acrostichum speciosum* had relatively more gene losses, with 7 (*psbF*, *rpl2*, *rpl21*, *ycf2*, *ycf12*, *ycf94*, and *trnT*-*UGU*) and 9 (*psbF*, *rpl2*, *rps11*, *ycf1*, *ycf2*, *ycf12*, *ycf94*, *trnR*-*UCG*, and *trnT*-*UGU*) missing genes, respectively. In addition, *trnR*-*UCG* and *trnT*-*UGU* were frequently absent in the 41 Pteridaceae cpDNAs, while *ycf94* showed a phenomenon of universal loss.

**Table 1:** CpDNA features of the 41 Pteridaceae species

Subfamily	Name	Size (bp)	GC%	Length (bp)			Accession No.
				LSC	IR	SSC	
Vittarioideae	*Adiantum aleuticum*	157,519	45.3	82,785	53,138	21,596	NC_040209.1
	*Adiantum capillus-veneris*	150,568	42.0	82,282	46,894	21,392	NC_004766.1
	*Adiantum flabellulatum*	152,063	43.3	83,384	47,230	21,449	NC_064144.1
	*Adiantum malesianum*	154,671	42.6	89,030	44,154	21,487	NC_063331.1
	*Adiantum nelumboides*	149,956	42.8	83,281	45,192	21,483	NC_050350.1
	*Adiantum reniforme* var. *sinense*	150,102	42.8	83,267	45,376	21,459	NC_062433.1
	*Adiantum shastense*	150,414	44.3	82,113	46,762	21,539	NC_037478.1
	*Adiantum tricholepis*	150,470	42.5	82,606	46,403	21,461	NC_040172.1
	*Antrophyum semicostatum*	150,274	40.1	87,392	42,054	20,828	NC_040176.1
	*Haplopteris elongata*	156,002	40.1	80,810	54,376	20,816	NC_040215.1
	*Scoliosorus ensiformis*	145,327	40.0	82,358	42,156	20,813	NC_040218.1
	*Vaginularia trichoidea*	147,102	39.2	84,017	43,155	19,930	NC_040175.1
	*Vittaria appalachiana*	149,531	40.1	84,330	44,370	20,831	NC_040219.1
	*Vittaria graminifolia*	151,035	40.1	86,058	44,132	20,845	NC_040217.1
Pteridoideae	*Gastoniella chaerophylla*	148,099	40.3	81,915	44,646	21,538	NC_040210.1
	*Onychium japonicum*	150,156	41.2	82,290	46,838	21,028	NC_040205.1
	*Pityrogramma trifoliata*	148,156	40.0	82,321	44,930	20,905	NC_040207.1
	*Pteris arisanensis*	160,191	42.4	81,989	57,086	21,116	NC_083994.1
	*Pteris ensiformis*	148,985	41.7	81,778	46,094	21,113	NC_083995.1
	*Pteris multifida*	153,916	42.2	82,027	50,760	21,129	NC_058883.1
	*Pteris semipinnata*	162,270	42.3	81,963	59,182	21,125	NC_060734.1
	*Pteris vittata*	154,106	41.7	82,602	50,550	20,954	MH173082.1
	*Taenitis blechnoides*	157,301	40.4	88,369	47,996	20,936	NC_083996.1
	*Tryonia myriophylla*	156,327	40.0	87,296	48,224	20,807	NC_040208.1
Parkerioideae	*Acrostichum speciosum*	156,095	38.4	84,476	49,734	21,885	NC_053768.1
	*Ceratopteris cornuta*	149,424	36.7	83,623	44,574	21,227	MH173068.1
	*Ceratopteris thalictroides*	149,399	36.7	83,580	44,577	21,241	NC_062137.1
Cryptogrammoideae	*Coniogramme intermedia*	153,561	45.0	82,817	49,508	21,236	NC_057002.1
	*Cryptogramma acrostichoides*	150,162	42.3	83,763	45,231	21,168	NC_040211.1
	*Llavea cordifolia*	149,387	41.9	81,944	46,416	21,027	NC_040216.1
Cheilanthoideae	*Bommeria hispida*	156,749	42.6	82,491	46,284	27,974	NC_040206.1
	*Calciphilopteris ludens*	157,068	43.5	82,423	53,170	21,475	NC_040214.1
	*Cheilanthes micropteris*	157,257	41.4	88,145	46,550	22,562	NC_040174.1
	*Hemionitis subcordata*	165,631	42.8	82,607	61,842	21,182	NC_040173.1
	*Myriopteris covillei*	155,548	42.7	83,079	51,148	21,321	NC_039724.1
	*Myriopteris lindheimeri*	155,770	42.7	83,059	51,388	21,323	NC_014592.1
	*Myriopteris scabra*	162,051	42.1	82,874	54,230	24,947	NC_040213.1
	*Notholaena standleyi*	159,556	42.4	83,769	54,522	21,265	NC_040203.1
	*Paragymnopteris bipinnata* var. *bipinnata*	150,736	42.5	82,926	46,516	21,294	NC_061171.1
	*Pellaea truncata*	150,713	42.5	82,865	46,480	21,368	NC_040202.1
	*Pentagramma triangularis*	153,445	41.8	85,668	46,763	21,014	NC_040171.1

LSC: Large-single copy, SSC: Small-single copy, IR: Inverted repeat

### Sequence variation analysis

Multiple alignments of the 41 Pteridaceae cpDNAs revealed higher divergence in non-coding sequences than in coding regions (Figure S2). Particularly, IGS regions exhibited significant differentiation, while coding regions like *matK*, *cemA*, *rpoC2* and *ycf1* also showed variation. Overall, the IR region of the 41 Pteridaceae cpDNAs had the highest degree of conservation, while the single copy region had less conservation. Nucleotide diversity (Pi) values ranged from 0.006 to 0.376 for common genes and from 0 to 0.603 for common IGS regions. *MatK*, *ndhF*, *ndhH* - *rps15*, and *trnL* - *ccsA* showed notably higher Pi values, indicating substantial single nucleotide polymorphism (Figure S3). These markers could be utilized for distinguishing different species or populations, with *matK* already recognized as the core DNA barcode for ferns [32].

### Repetitive sequence analyses

**Fig. 1:**
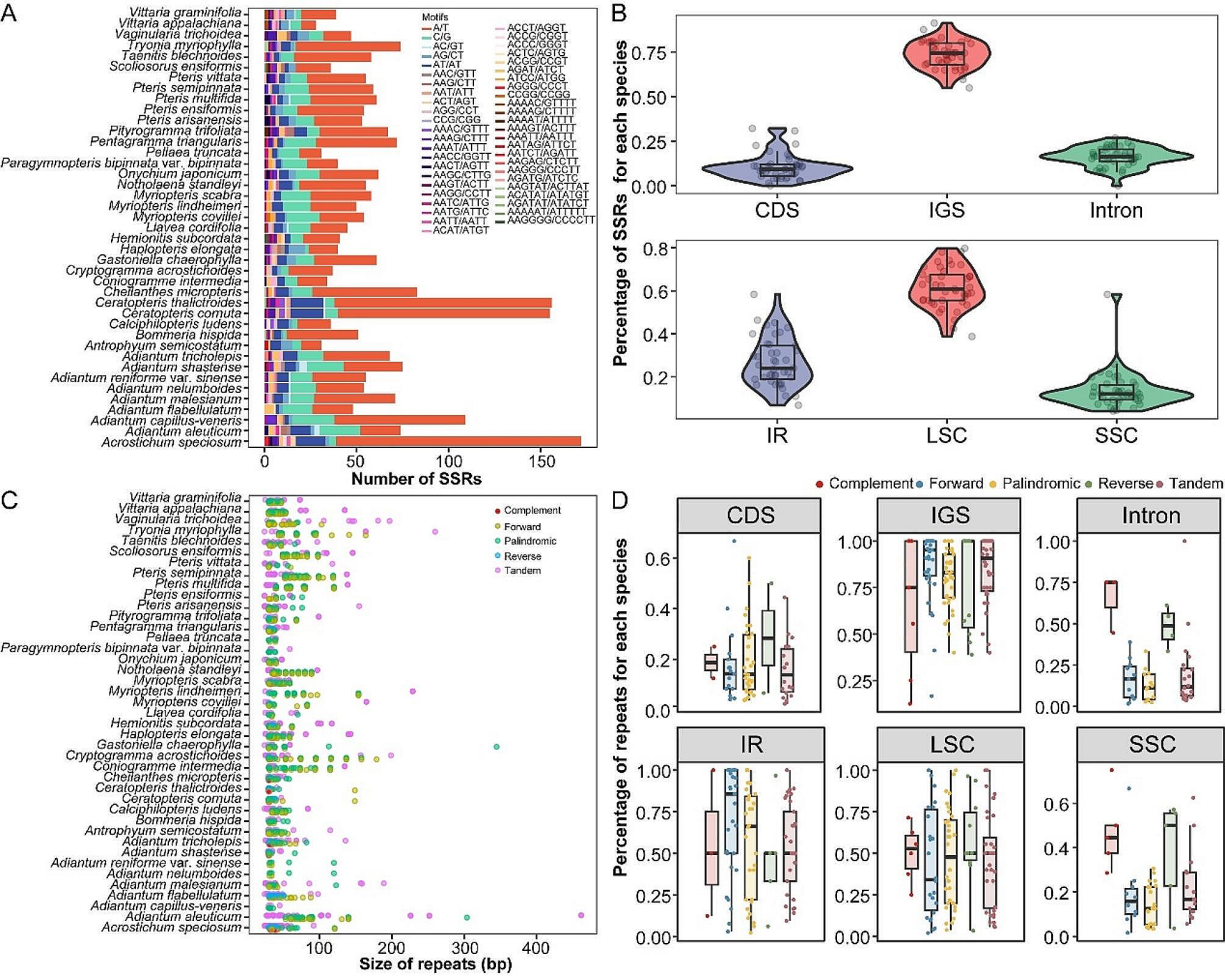
Comparison of repetitive sequences among the 41 Pteridaceae cpDNAs. **(A)** The number of SSRs among each species. **(B)** The percentage of SSRs located in different cpDNA regions and gene sequence regions. **(C)** The size distribution of dispersed repeats and tandem repeats among the 41 Pteridaceae cpDNAs. **(D)** The percentage of dispersed repeats and tandem repeats located in different cpDNA regions and gene sequence regions

The number of simple sequence repeats (SSRs) in the 41 Pteridaceae cpDNAs ranged from 28 in *Vittaria appalachiana* to 172 in *A. speciosum* (Fig. 1A, Table S1). A/T motifs, especially in *C. cornuta*, *A. speciosum*, and *C. thalictroides*, dominated the SSR motifs. Hexanucleotide repeats were the least common, accounting for 0.64% (*C. thalictroides*) to 4.88% (*H. subcordata*). SSRs were predominantly located in the LSC region (median: 60.81%), followed by the IR regions (median: 24%) and SSC region (median: 12.05%) (Fig. 1B, Table S1). In comparison to the CDS regions (median: 9.26%) and intron regions (median: 16.36%), most SSRs were found in the IGS regions (median: 74.36%) (Fig. 1B, Table S1).

Dispersed repeats, predominantly forward and palindromic, were found in the cpDNAs of all 41 Pteridaceae species, with complement and reverse repeats observed in a few species (Table S2). Tandem repeats were identified in all other species except *Adiantum nelumboides* and *Adiantum reniforme* var. *sinense* (Table S3). Most repeats were within 100 bp, with some exceeding 200 bp (Fig. 1C). The majority of repeats were in the LSC (median: 48.80%) and IR regions (median: 54.21%), compared to the SSC region (median: 7.84%) (Fig. 1D). Additionally, repeats are more prevalent in the IGS regions (median: 86.96%) compared to the CDS (median: 12.01%) and intron regions (median: 4.41%) (Fig. 1D).

### Expansion and contraction of IR boundary analysis

**Fig. 2:**
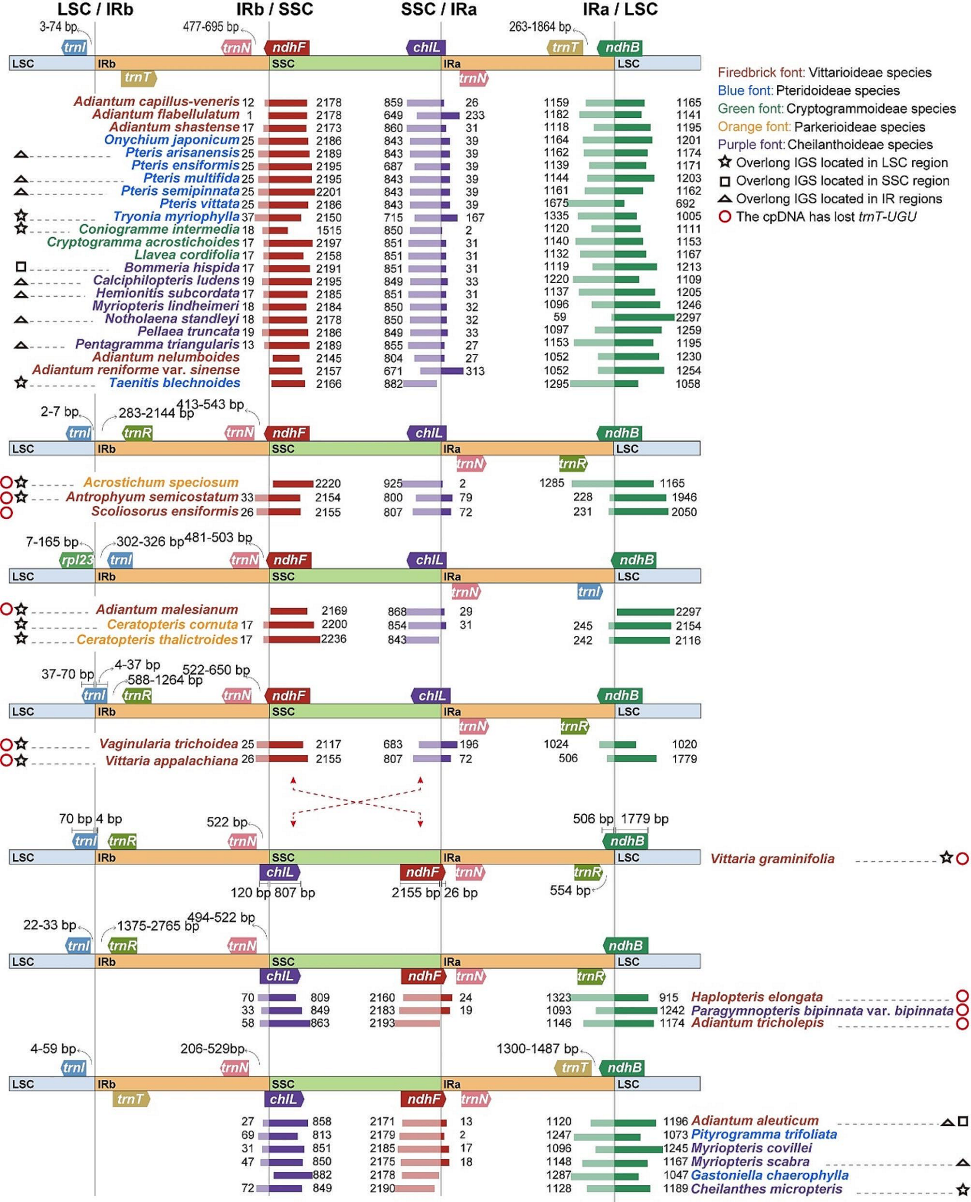
Comparison of IR/SC boundaries among the 41 Pteridaceae cpDNAs. The numbers above, below, or adjacent to genes represent gene length or the distances from the front or end of genes to the boundary sites. Figure features are not to scale

The IR/SC boundary genes of the 41 Pteridaceae species had varying degrees of expansion and contraction. No similar patterns were found among the IR/SC boundaries in five different subfamilies (Fig. 2). The genes located at the IR/SC boundaries of these species, primarily included *rpl23*, *trnI-CAU*, *trnT-UGU*, *trnR-ACG*, *ndhF*, *chlL*, *trnN-GUU*, and *ndhB*. The IR/SSC boundary genes in these species were consistent, with only slight displacement near the boundary. The main reason for the differences was the inversion of the SSC region. In contrast, the LSC/IR boundary underwent much greater changes, such as *trnI-CAU* of *Adiantum malesianum*, *Ceratopteris cornuta*, *Ceratopteris thalictroides* and *Vaginularia trichoidea* all entering the IRb region; while the *trnI-CAU* of other species was located in the LSC region or on the LSC/IRb boundary. Another reason for differences in IR/LSC boundary genes was the absence of *trnT-UGU* in some species.

### The relationship between overlong IGS and CpDNA size

**Fig. 3:**
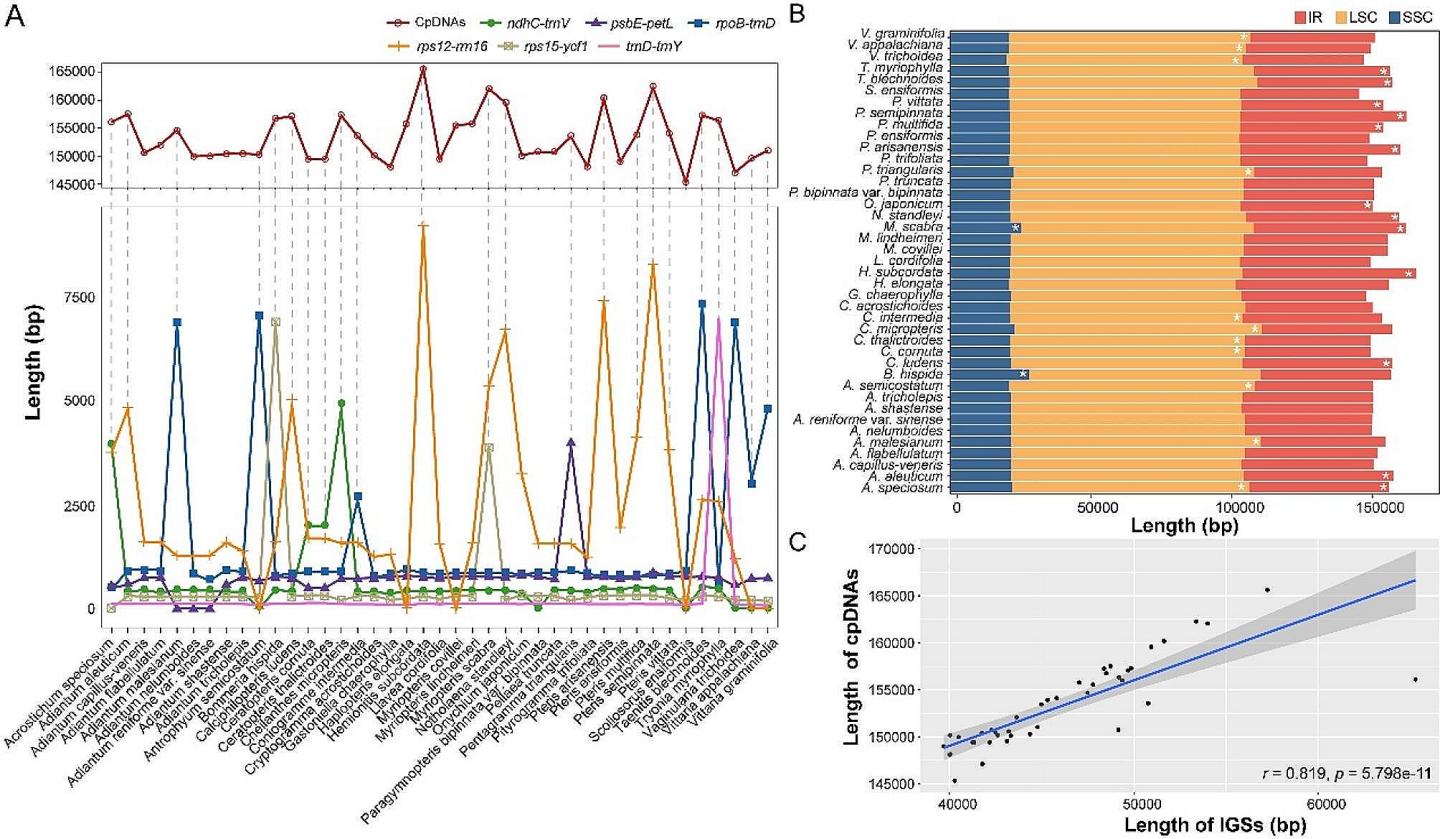
Comparison of chloroplast genome features in the 41 Pteridaceae species. **(A)** Comparison of the cpDNA sizes with the overlong IGSs; even if the overlong IGS is in the IR regions, the figure only shows the length of one copy of the IGS. **(B)** Comparison of the lengths of the LSC, SSC, and IR regions; with * indicating regions containing overlong IGS. **(C)** The correlation between IGS length and cpDNA sizes

Overlong IGSs seemed to be common in the Pteridaceae cpDNAs, but no reliable patterns of occurrence were found between subfamilies or genera (Fig. 2). In this study, within the same IGS across different species, lengths greater than the mean of these IGSs were defined as overlong IGS. By comparing the length of the positions with overlong IGS in the Pteridaceae cpDNAs (Table 2), it was found that they frequently occurred in the *rpoB - trnD* region of the LSC and the *rps12 - rrn16* region of the IRs. Additionally, in some species, the *trnD - trnY* (LSC), *ndhC- trnV* (LSC), *psbE - petL* (LSC), and *rps15 - ycf1* (SSC) IGSs also had longer lengths. Chloroplast genes were usually relatively conservative, so this random change of overlong IGSs was likely the main reason for the difference in cpDNA sizes of Pteridaceae species (Fig. 3A). For instance, *Hemionitis subcordata*, *Myriopteris scabra*, and *Pteris semipinnata* had larger cpDNAs, and their interiors contained overlong IGS regions. The lengths of the LSC, SSC, and IR regions of these species were separately calculated, and it was found that most of the factors contributing to the differences in these region lengths were largely due to the presence of these overlong IGSs (Fig. 3B). Moreover, the length of IGSs in each species was linearly related to their cpDNA sizes (Fig. 3C), and there was a highly significant positive correlation (*r* = 0.819, *p* = 5.798e-11 < 0.001).

**Table 2:** Comparison of overlong IGSs in the 41 Pteridaceae cpDNAs

Species	rpoB-trnD (bp)	trnD-trnY (bp)	ndhC-trnV (bp)	psbE-petL (bp)	rps12-rrn16 (bp)	rps15-ycf1 (bp)	cpDNA size
*Adiantum aleuticum*	932	110	402	566	**4785**	305	157,519
*Adiantum capillus-veneris*	944	114	448	740	1600	290	150,568
*Adiantum flabellulatum*	935	114	380	749	1593	289	152,063
*Adiantum malesianum*	**6877**	116	447	-	1265	295	154,671
*Adiantum nelumboides*	833	124	433	-	1260	292	149,956
*Adiantum reniforme* var. *sinense*	669	124	433	-	1270	292	150,102
*Adiantum shastense*	929	119	402	568	1584	293	150,414
*Adiantum tricholepis*	878	110	405	765	1378	286	150,470
*Antrophyum semicostatum*	**7061**	92	-	664	-	279	150,274
*Haplopteris elongata*	944	87	419	797	-	232	156,002
*Scoliosorus ensiformis*	901	99	-	757	-	208	145,327
*Vaginularia trichoidea*	**6880**	90	-	537	1200	211	147,102
*Vittaria appalachiana*	**3067**	95	-	723	-	201	149,531
*Vittaria graminifolia*	**4817**	72	-	735	-	183	151,035
*Gastoniella chaerophylla*	826	99	360	751	1301	209	148,099
*Onychium japonicum*	820	106	379	807	**3221**	332	150,156
*Pityrogramma trifoliata*	831	108	394	751	1237	273	148,156
*Pteris arisanensis*	779	105	493	747	**7391**	320	160,191
*Pteris ensiformis*	778	105	461	700	1923	321	148,985
*Pteris multifida*	773	111	518	744	**4076**	325	153,916
*Pteris semipinnata*	783	105	498	873	**8272**	321	162,270
*Pteris vittata*	826	106	454	776	**3549**	243	154,106
*Taenitis blechnoides*	**7346**	113	530	766	2617	310	157,301
*Tryonia myriophylla*	584	**6943**	493	750	2564	294	156,327
*Acrostichum speciosum*	443	116	**3953**	503	**3730**	-	156,095
*Ceratopteris cornuta*	879	134	**1984**	492	1684	322	149,424
*Ceratopteris thalictroides*	886	133	**1984**	491	1683	322	149,399
*Coniogramme intermedia*	**2593**	105	393	725	1590	305	153,561
*Cryptogramma acrostichoides*	744	105	397	741	1241	306	150,162
*Llavea cordifolia*	818	103	416	721	1563	229	149,387
*Bommeria hispida*	793	118	447	765	1581	**6872**	156,749
*Calciphilopteris ludens*	846	119	416	752	**4874**	301	157,068
*Cheilanthes micropteris*	875	109	**4760**	722	1568	199	157,257
*Hemionitis subcordata*	851	119	422	758	**9179**	304	165,631
*Myriopteris covillei*	858	125	440	765	1570	306	155,548
*Myriopteris lindheimeri*	865	121	453	769	1567	306	155,770
*Myriopteris scabra*	849	122	438	761	**5336**	**3877**	162,051
*Notholaena standleyi*	863	113	445	751	**6635**	199	159,556
*Paragymnopteris bipinnata* var. *bipinnata*	862	133	-	771	1565	303	150,736
*Pellaea truncata*	862	124	461	694	1567	305	150,713
*Pentagramma triangularis*	908	114	443	**3890**	1558	194	153,445

*trnD*: *trnD-GUC*; *trnY*: *trnY-GUA*; *trnV*: *trnV-UAC*. The presence of “-” in the data indicates the presence of corresponding gene loss in cpDNAs. Bold values represents overlong IGS

### Characteristics and analysis of overlong IGSs

**Fig. 4:**
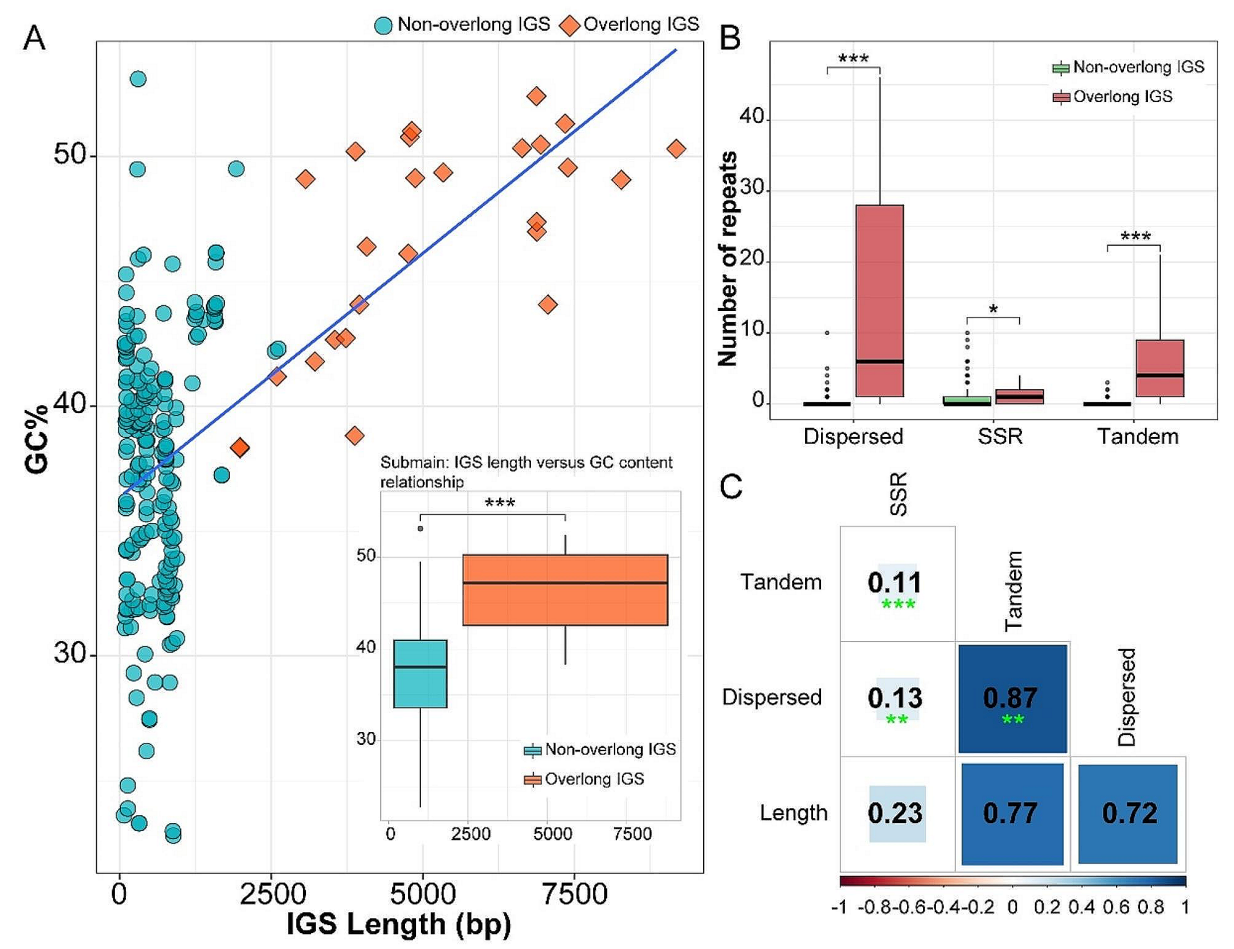
Comparison of **(A)** GC content and **(B)** number of repeats between overlong and non-overlong IGSs. **(C)** Correlation among SSRs, tandem repeats, dispersed repeats, and the length of overlong IGSs. *p < 0.05; **p < 0.01; ***p < 0.001

This study analyzed the GC content of these sequences and found that the overall GC content of cpDNAs was less affected by these overlong IGSs. However, specific IGSs encompassing both overlong and non-overlong situations exhibited a significant difference (*p =* 1.189e-11 < 0.001); overlong IGSs tended to exhibit higher GC content (Fig. 4A). These expanded IGSs exhibited collinearity across diverse intergenic regions in various species (Figure S4). Upon conducting homologous sequence alignment of these elongated IGSs in the NCBI database, it was found that the majority of these homologous sequences originated from fern cpDNAs. In certain overlong IGSs, such as *rps12-rrn16*, alignments were observed with sequences from mitochondrial genomes of *Haplopteris ensiformis* (Pteridaceae), suggesting that they may transfer within organelles through mechanisms such as gene transfer or horizontal gene transfer. In addition, repetitive sequences and transposable elements located within these IGS were screened. The results of the Mann-Whitney U test revealed that compared to non-overlong IGSs, there were significantly higher numbers of SSRs (*p* = 0.016), tandem repeats (*p* = 2.2e-16 < 0.001), and dispersed repeats (*p* = 2.2e-16 < 0.001) in overlong IGSs (Fig. 4B). Regarding the length relationship between repetitive sequences and IGSs, although not statistically significant, a strong positive correlation was observed between tandem repeats and dispersed repeats with the expansion of the IGSs (*r* = 0.77 and 0.72, respectively) (Fig. 4C). For transposable elements, relevant sequences could not be retrieved structurally, but similar short fragments of different types of transposable elements were identified in *A. malesianum* (*Gypsy*, 48 bp) and *Pteris arisanensis* (*Copia*, 65 bp) (Table 3).

**Table 3:** The homologous fragments of transposable elements contained in the overlong IGSs

Species	Overlong IGS	Start-End	Matching repeat	Repeat class/family	Start-End
*Adiantum malesianum*	*rpoB - trnD*	28,584–28,631	Gypsy-14_SB-I	LTR/Gypsy	2773–2821
*Pteris arisanensis*	*rrn16 - rps12*	93,518–93,582	Copia-7_Mad-I	LTR/Copia	6358−6295
*Pteris arisanensis*	*rps12 - rrn16*	148,599–148,663	Copia-7_Mad-I	LTR/Copia	6295–6358

### Phylogenetic relationship and divergence time estimate

**Fig. 5:**
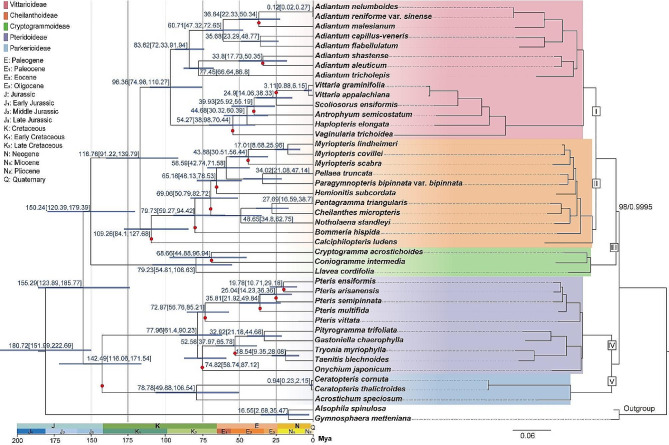
Phylogenetic relationship (right) and divergent time estimate (left) of the 41 Pteridaceae species. The mean divergence time of the nodes is shown next to the nodes while the blue bars correspond to the 95% highest posterior density (HPD). The red dots represent species within the branch that contain overlong IGS. Bootstrap value/posterior probabilities < 100%/1 are displayed on the branches

The BI tree and ML tree, constructed using the common protein-coding sequences of all species, were consistent (Fig. 5). Reconstructed phylogenetic relationships received high support, with the lowest node support being 98%. Here, Pteridaceae species were divided into five subfamily clades: clade I (Vittarioideae), clade II (Cheilanthoideae), clade III (Cryptogrammoideae), clade IV (Pteridoideae), and clade V (Parkerioideae). Their divergence from the outgroup could be traced back to the Jurassic period (∼ 180.72 Mya). Clades I, II, and III share a more recent common ancestor, indicating a closer phylogenetic relationship; this common ancestor diverged in the Late Jurassic period, approximately 155.29 Mya. The common ancestor of clades I and II further diverged around 150.24 million years ago in the same period. Additionally, clades I and II diverged during the Early Cretaceous period (∼ 116.76 Mya). Clades IV and V shared a common ancestor dating back to approximately 142.49 Mya, near the Jurassic-Cretaceous (J/K) boundary. The phylogenetic tree strongly supported *Pteris* and *Adiantum* as monophyletic clades, and both of their ancestral clades diverged during the Late Cretaceous period, during which most genera of the fern family began to rapidly differentiate. Overlong IGSs were present during the early divergence of the family, and as species rapidly diversified, this type of overlong IGSs gradually became more prevalent.

## Discussion

This study sequenced the cpDNA structures of five Pteridaceae species and examined those of 41 species, covering all subfamilies. They exhibited typical quadripartite structures, with genome sizes ranging from 145,327 bp to 165,631 bp and GC contents between 36.7% and 45.3%. Upon re-aligning and completing missing gene annotations, higher gene losses were observed in *P. bipinnata* var. *bipinnata* (7 chloroplast genes lost) and *A. speciosum* (9 lost) among the Pteridaceae cpDNAs. Additionally, *trnR-UCG* and *trnT-UGU* were frequently lost among the 41 Pteridaceae cpDNAs, along with a common loss of *ycf94*. The alignment of all 41 Pteridaceae cpDNAs revealed poor alignment in non-coding regions, especially in the IGS regions. Four regions with significantly higher Pi values compared to other genes/IGSs were identified: *matK*, *ndhF*, *ndhH - rps15*, and *trnL - ccsA*. Among these, *matK* has been used as a core DNA barcode for ferns, and the other three markers may serve as candidate DNA barcodes for species within the Pteridaceae family.

Repetitive sequences can be dispersed widely or found in simple tandem arrays. SSRs, also known as microsatellites, consist of 1–6 nucleotide tandem repeat motifs and are distributed throughout the genome [33, 34]. SSRs are highly polymorphic and specific, making them valuable for studying molecular evolution, genetic diversity, and developing molecular markers [35, 36]. The diversity in repeat length, copy number, and distribution within species is attributed to slipped-strand mispairing during DNA replication on a single strand [37, 38]. Mononucleotide repeats, especially A/T motifs, were the most common in this study. A potential reason for the higher frequency of A/T repeats is that during chloroplast genome replication, the separation of AT strands is relatively easier compared to GC, which increases slip mismatching [39]. In the Pteridaceae cpDNAs, SSRs were mainly located in the LSC region (38.71–79.73%) and tended to occur in IGS (54.84–91.18%), possibly due to stronger constraints in coding regions [40]. Short repeat units can also be further extended into longer tandem repeats through slipped-strand mispairing or recombination [41–43], with the number of tandem repeats varying due to susceptibility to slippage events during DNA replication [44]. Dispersed repeats are often associated with and contribute significantly to the chloroplast genome rearrangement in plants [25, 45]. Here, all species except *A. nelumboides* and *A. reniforme* var. *sinense* exhibited tandem and dispersed repeats, primarily less than 100 bp in size, with forward and palindromic repeat motifs predominating. Furthermore, these repeats were more prevalent in the IGS regions.

The substitution rate in chloroplast IR region genes is significantly lower than that in the SC region, thus greater conservation in the IR region [46]. However, structural variations in the IR/SC boundary regions are still common [47–49]. Among the 41 Pteridaceae cpDNAs, varying degrees of IR/SC boundary expansion and contraction were observed, even within the same genus. The IR/SSC boundary genes remained consistent in the Pteridaceae cpDNAs, with differences primarily attributed to SSC region inversions. In contrast, the LSC/IR boundary varied more due to changes in the *trnI-CAU* position and the absence of *trnT-UGU* in some species. The variation in cpDNA size is often associated with changes in the IR/SC boundary [50–52] and the expansion of repetitive sequences [30, 31]. In this study, the movement in the IR/SC boundary genes of Pteridaceae cpDNA only led to minor differences in cpDNA size. For instance, the cpDNA size of *H. subcordata* was the largest, with a longer IR region due to the expansion of the *rps12 - rrn16* IGS, rather than significant expansion of its IR/SC boundaries. A strong correlation between cpDNAs and IGSs was observed, and there was a common occurrence of overlong IGSs in species within this family. These overlong IGSs consistently aligned with the changes in the Pteridaceae cpDNA size, implying their primary influence on cpDNA size and their potential role in driving cpDNA structure evolution [53]. In cases like *A. malesianum*, overlong IGS amplifies cpDNA size and triggers sequential movement of LSC region genes, affecting IR/SC boundaries [48].

The overlong IGSs prevalent in the Pteridaceae family were found in various intergenic regions across different species and showed a degree of collinearity (Figure S4). Mobile elements are present in the fern cpDNAs and are often found near genome inversion sites [53]. In this study, only a few inversions occurred in the Pteridaceae cpDNAs, such as the *ndhJ - psbE*, the *rrn5 - rrn16*, and the SSC region. Some overlong IGSs were also found near inversion sites, such as *rrn16 - rps12*, which may have served as hotspots for IGS expansion. Additionally, the *psbE - petL* IGS of *Pentagramma triangularis* also underwent expansion. Within the same IGS, the GC content of overlong IGSs that underwent expansion was consistently higher, showing a significant difference compared to non-overlong IGSs. An important characteristic of GC base pairs is their higher thermal stability compared to AT base pairs [54]. These interactions appear to be crucial for the overall structural stability of DNA and RNA transcripts [55, 56]. Significant differences in GC content exist among different genomes and within different regions of genomes. Some studies suggest a correlation between GC content and the length of coding genes, where the length of exons often increases with higher GC content [57]. This is because stop codons are rich in AT, consequently resulting in a lower frequency of stop codon occurrence in GC-rich exons [58]. The increase in GC content may also be attributed to the presence of more GC-rich sequences within these overlong IGSs, such as repetitive sequences. In the Pteridaceae cpDNAs, the overlong IGSs contained significantly more repetitive sequences, especially tandem repeats and dispersed repeats; meanwhile, these repeats had a strong positive correlation with the expansion of IGSs (*r* = 0.77 and 0.72, respectively), although statistical significance was not achieved. This suggests that repetitive sequences may promote the occurrence of chloroplast genome structural variation (SV). For instance, the location of SV in the *Carex* cpDNAs is closely related to the location of long repeats [59]. The amplification of the *Cyripedium* cpDNAs is associated with a surge in AT-biased repeats [30]. In addition, similar fragments were observed in the mitochondrial genomes of *H. ensiformis* and detected transposable element-like fragments in a few species, suggesting that they may transfer among different organelles.

According to this study, the Pteridaceae family had clear boundaries in both subfamilies and genera. The *Pteris* and *Adiantum* were both monophyletic, consistent with previous research [2, 60–62]. Based on fossil evidence, ferns are believed to have originated in the Devonian period [63], and their dominance continued into the Paleozoic era [64]. Here, the MCMCTree model suggested that the divergence of the Pteridaceae family from the outgroup occurred during the Jurassic period (∼ 180.72 Mya). Fossil records from the Jurassic period indicate significant fern evolution [65, 66], with favorable climate and environmental conditions contributing to their survival and reproduction during this time. As a result, ferns occupied a crucial ecological niche on Earth during this period, evolving a wide range of morphological and ecological characteristics that had a significant impact on the evolution and diversity of terrestrial ecosystems [67]. The J/K boundary period represents a time of environmental upheaval, characterized by intense transgressive phases due to rapidly changing sea levels [68]. The subfamily of Parkerioideae represents an aquatic branch, with its species thriving in wet aquatic environments [69, 70], diverged around the J/K boundary period (∼ 142.49 Mya) and possibly underwent adaptive evolution. In addition, the divergence of the genus *Acrostichum*, within Parkerioideae, occurred around the Late Cretaceous (∼ 78.78 Mya), overlapping with the fossil record of this genus [71]. During the Late Cretaceous period, the genus of this family began to rapidly diverge. Overlong IGSs were present during the early divergence stages of the Pteridaceae family, indicating that this structural feature may have an ancient origin in related species. As species rapidly diversified, the prevalence of these overlong IGSs gradually increased.

## Conclusion

This study offers comprehensive insights into Pteridaceae cpDNAs. Chloroplast gene numbers were mostly stable, except for *P. bipinnata* var. *bipinnata* and *A. speciosum*. Changes in LSC/IR boundaries resulted from *trnI-CAU* movement and *trnT-UGU* deletion. SSC/IR boundary shifts were mainly due to SSC region inversion. The Pteridaceae cpDNAs often had overlong IGSs, increasing non-coding region variability and affecting cpDNA size changes. These overlong IGSs had higher GC content and were rich in repetitive sequences. Divergence time analysis traced Pteridaceae separation to the Jurassic (∼ 180.72 Mya), with rapid diversification within the genera beginning in the Late Cretaceous period. Additionally, overlong IGSs may have already existed during the early differentiation stages of this family. This study provides further theoretical support for the classification of Pteridaceae species, genetic diversity, and the evolution of genomic structure.

## Materials and methods

### Plant materials, DNA extraction and De Novo sequencing

Obtaining a more complete chloroplast genome helps to understand their structural evolution. This study added cpDNAs of species *Pteris ensiformis*, *P. arisanensis*, *Taenitis blechnoides*, *Adiantum flabellulatum* and *A. malesianum.* Fresh leaves of the first three were sampled from the campus of Shenzhen Fairy Lake Botanical Garden [72]. Fresh leaves of the latter two were sampled from the campus of South China Agricultural University (SCAU) [48]. The plant materials used in the study were identified by Ting Wang and deposited in the Herbarium of SCAU with specimen numbers GXL20210901 (*A. flabellulatum*), GXL20210902 (*A. malesianum*), GXL20210903 (*P. ensiformis*), GXL20210904 (*P. arisanensis*), and GXL20210905 (*T. blechnoides*). DNA was extracted from the samples using a Tiangen Plant Genome DNA Kit (Tiangen Biotech Co., Ltd., Beijing, China) according to the manufacturer’s instructions. The Illumina NovaSeq6000 platform was used for sequencing.

### Sequence assembly and annotation

The complete cpDNAs were assembled using GetOrganelle [73] and Novoplasty [74]. NUMER [75] was used to check their collinearity. The cpDNAs were annotated by GeSeq [76] with *A. capillus-veneris* as the reference, and manually corrected. The cpDNAs were submitted to NCBI (National Center for Biotechnology Information) under GenBank accession numbers NC_083994.1 (*P. arisanensis*), NC_083995.1 (*P. ensiformis*), NC_083996.1 (*T. blechnoides*), NC_064144.1 (*A. flabellulatum*), and NC_063331.1 (*A. malesianum*).

### Comparative genome and boundary regions analysis

The complete cpDNAs of 36 Pteridaceae species were downloaded from GenBank (Table 1). Combining our five sequenced species, a total of 41 Pteridaceae species were examined, covering all subfamilies. The accuracy of gene annotation for these 41 Pteridaceae cpDNAs was rechecked, and local BLAST was used for homologous sequence retrieval to complete some missing annotation genes (Figure S1). The boundary region of the 41 Pteridaceae cpDNAs was rechecked using Geneious [77] and displayed using Adobe Illustrator 2020, to better observe the expansion/contraction of IR regions.

### Repetitive sequence analyses

The Perl script MISA (http://pgrc.ipk-gatersleben.de/misa/) [78] was used with the filter thresholds set to detect SSRs. The following parameters were set: a minimal repeat number of 10 for mononucleotide repeats, 5 for di-, 4 for tri-, and 3 for tetra-, penta-, and hexanucleotide SSRs. Tandem repeats were found with the tandem repeats finder (TRF) using default parameters [79]. To identify complex repetitive sequences such as forward, reverse, complement and palindromic, REPuter online software [80] was used with a minimum repeat size of 30 bp and 90% sequence identity (Hamming distance of 3). The transposable elements were retrieved using RepeatMasker [81], with the rmblast database selected and the reference species aligned to “viridiplantae”, with all other parameters set to default.

### Sequence divergence analysis

The comparative analysis was carried out by using the shuffle-LAGAN mode in mVISTA online tool [82] to analyze the cpDNA divergence of the 41 Pteridaceae species, with *A. capillus-veneris* (NC_004766.1) as a reference. Extracted the common genes and IGSs of these cpDNAs as independent datasets, aligned each dataset in MAFFT v7.475 using default parameters [83], and calculated nucleotide diversity in DnaSP v6.0 [84].

### Phylogenetic analysis and divergence time estimates

Phylogenetic reconstruction of the above 41 Pteridaceae species with *Gymnosphaera metteniana* and *Alsophila spinulosa* as outgroups. 76 common but not repetitive protein-coding sequences of these species were retained, and MAFFT was used to perform sequence alignment, and remove 90% of the gaps in each multi-alignment sequence using trimAl [85]. PhyloSuite [86] was used to concatenate these sequences into a dataset for phylogenetic analysis. The ML tree was inferred using RAxML [87], GTRGAMMAI was selected as the nucleotide substitution model. The Bayesian inference (BI) tree was established by MrBayes [88] and was estimated by running 2,000,000 generations (Nst = 6, rates = invgamma).

In this study, the differentiation time estimated by TimeTree [89] is used to calibrate the time tree (*A. aleuticum & Calciphilopteris ludens*: 57.8–129.7 Mya; *A. speciosum* & *A. spinulosa*: 154.7–228.8 Mya; *Pteris multifida* & *Pteris vittate*: 51.3–89 Mya; *A. nelumboides* & *Adiantum tricholepis*: 34.8–88.4 Mya). Inferring the time tree of Pteridaceae using the MCMCTree software package of PAML [90], the model was set to GTR, and the MCMC procedures had a burn-in of 2,000 iterations and then ran for 20,000 iterations. MCMCTree analysis was performed twice, which generated similar results, confirming the robustness of the analysis. The final tree was visualized and edited in FigTree v.1.4.3 (http://tree.bio.ed.ac.uk/software/figtree/).

### Electronic supplementary material

Below is the link to the electronic supplementary material.

Supplementary Material 1

Supplementary Material 2

## Data Availability

All data generated or analysed during this study are included in this published article and its supplementary information files.

## Declarations

### Appropriate permissions and/or licences for collection of plant or seed specimens

The plant materials in this study are from cultivated plants, and leaf collection has been approved by Shenzhen Fairy Lake Botanical Garden and South China Agricultural University.
