# Supplementary material for: The size diversity of the Pteridaceae family chloroplast genome is caused by overlong intergenic spacers
Source: BMC Genomics. 2024 Apr 22;25:396. doi: 10.1186/s12864-024-10296-0 (PMC11036588; doi:10.1186/s12864-024-10296-0)
Supplement: Supplementary file 1 — Supplementary Material 1 [file 12864_2024_10296_MOESM1_ESM.docx]

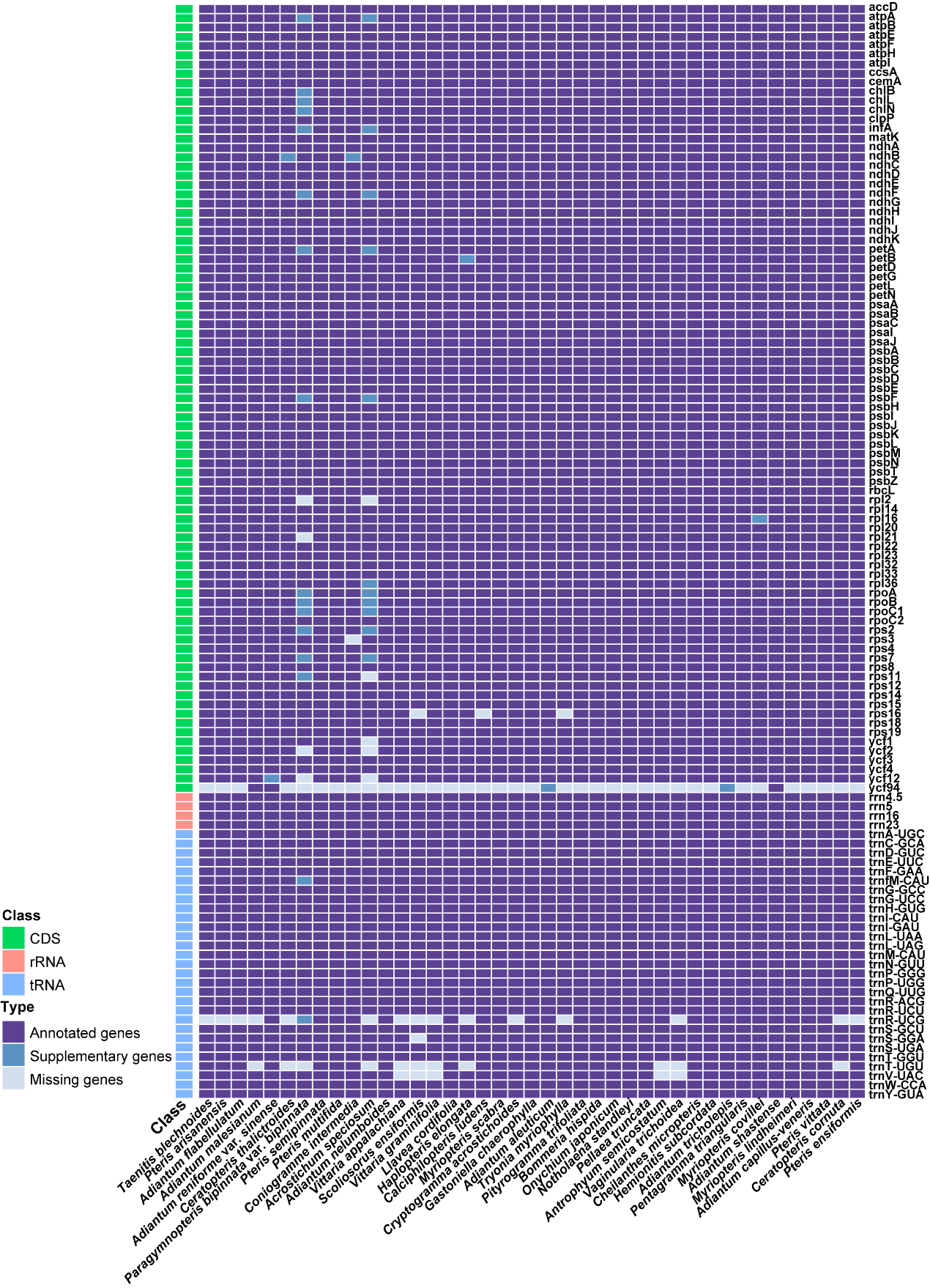


**Figure S1.** Gene deletion information of the Pteridaceae cpDNAs. We search for missing gene annotation in Pteridaceae cpDNAs using local BLAST and homologous sequences from each species. Using *Adiantum capillus-veneris* as the reference sequence for all species except itself. For *Adiantum capillus-veneris*, we select a reference sequence within the *Adiantum*.


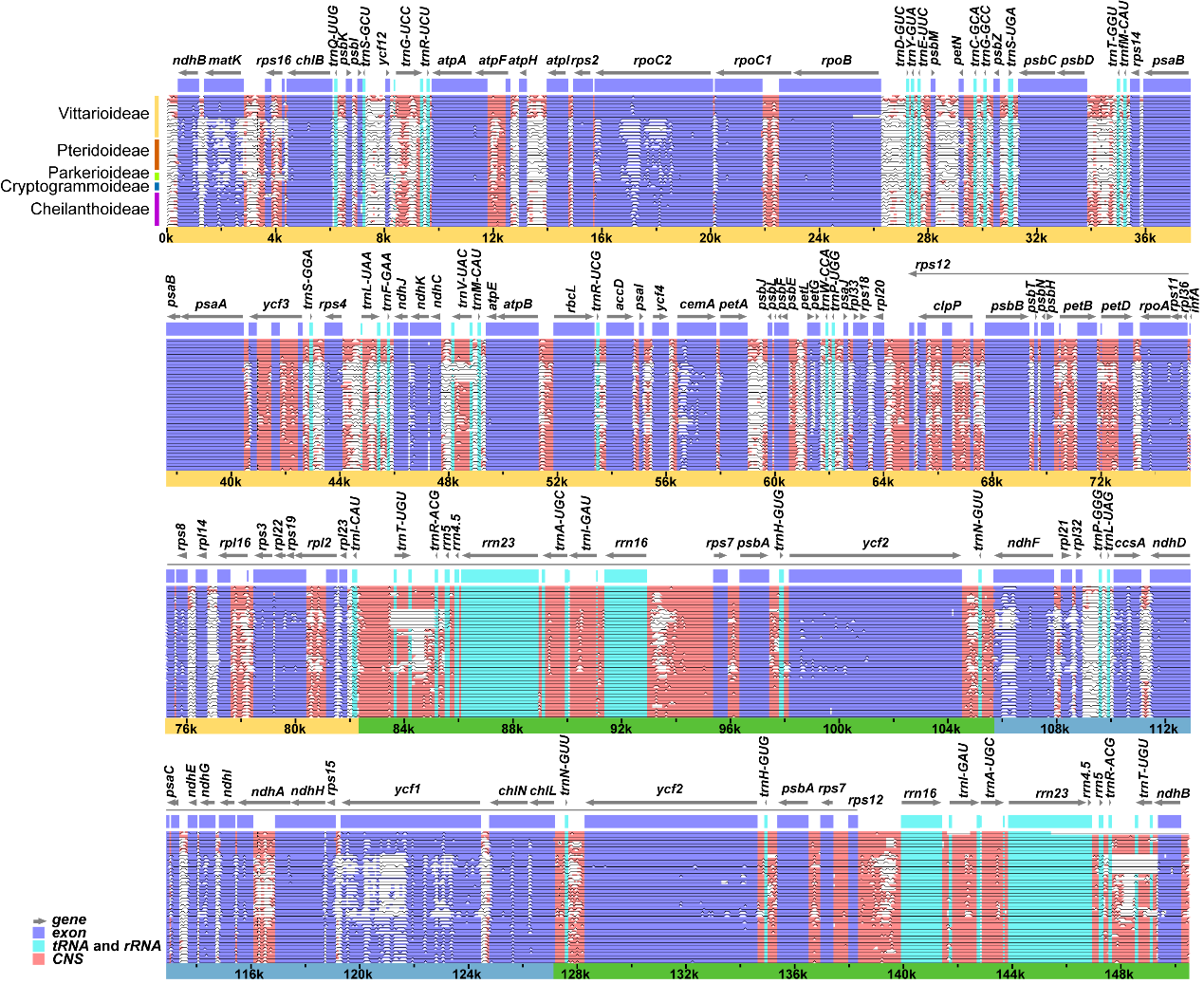


**Figure S2.** Global alignment of the 41 Pteridaceae cpDNAs using the *A. capillus-veneris* cpDNA as the reference.


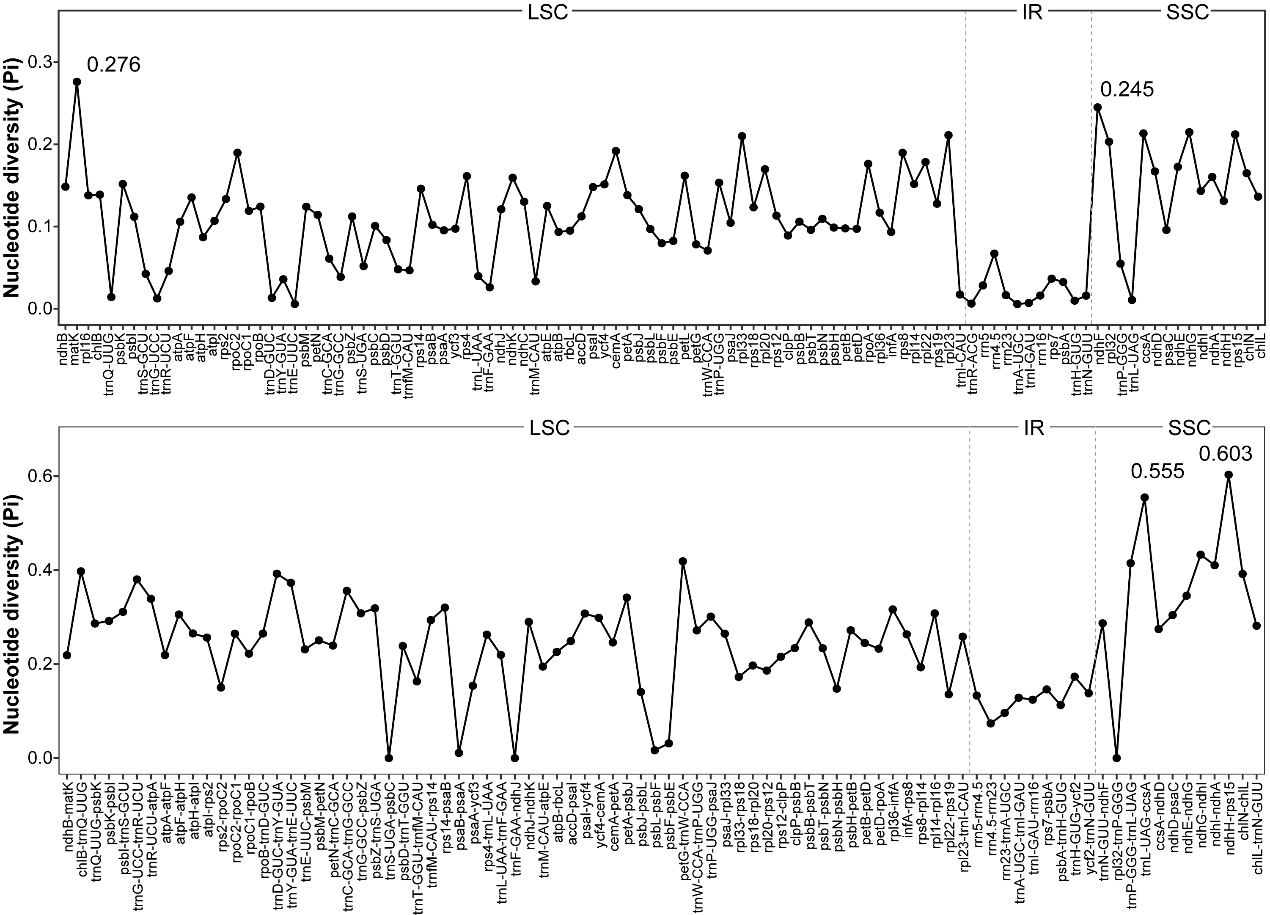


**Figure S3.** Nucleotide diversity analysis among the multiple alignments of the 41 Pteridaceae cpDNAs.


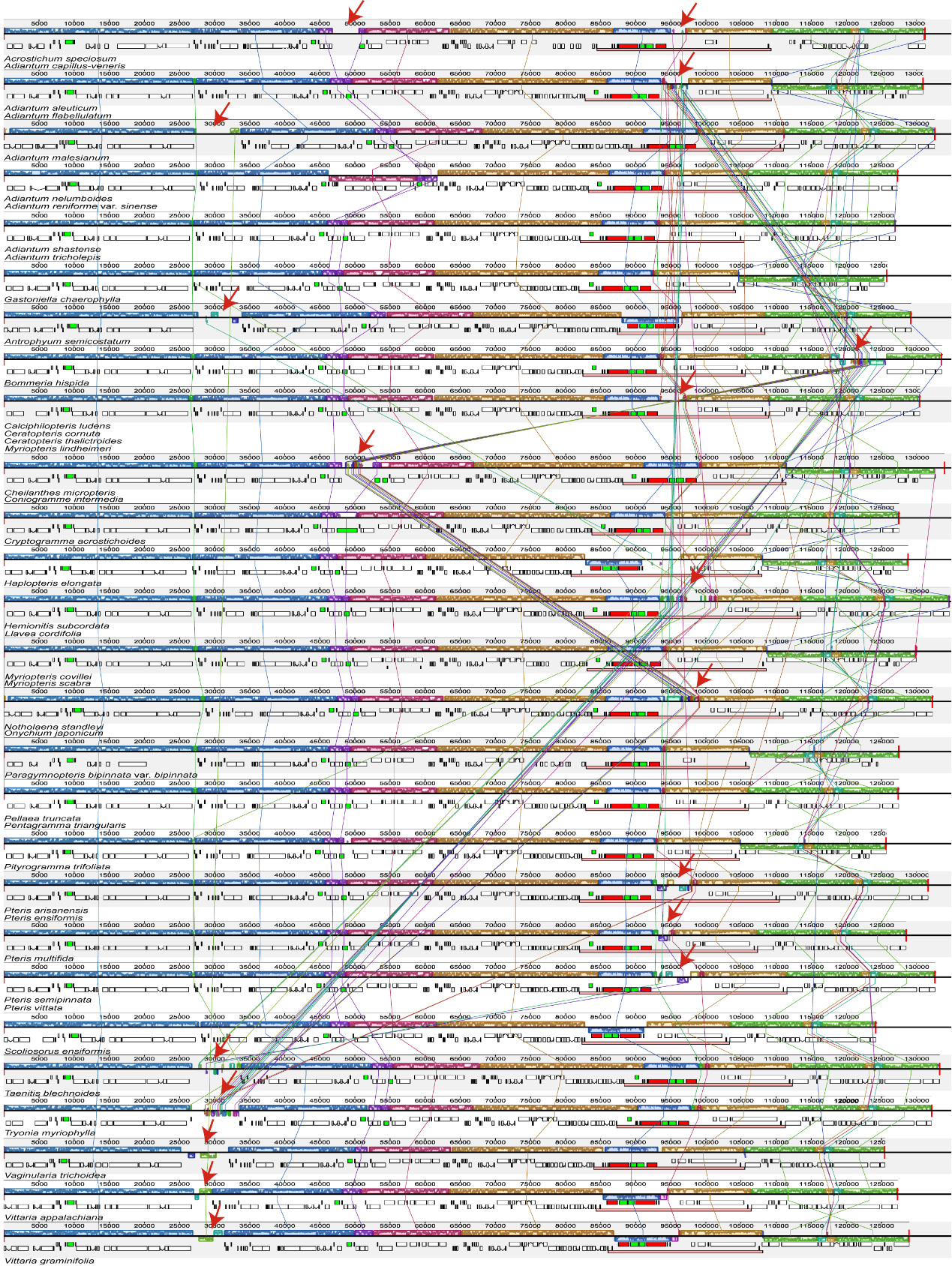


**Figure S4.** The collinearity of Pteridaceae cpDNAs. Species that have not undergone structural variation compared to the former are omitted for display; the red arrow indicates overlong IGS.
